# Fluctuating asymmetry in third molar agenesis as an aid to estimate socioeconomic status

**DOI:** 10.1007/s12024-023-00706-2

**Published:** 2023-09-06

**Authors:** Ana Rita Dinis, Alexandra Teixeira, Daniel Pérez-Mongiovi, Inês Morais Caldas

**Affiliations:** 1 1H-TOXRUN – One Health Toxicology Research Unit, University Institute of Health Sciences (IUCS), CESPU, CRL, 4585-116 Gandra, Portugal; 2 Faculty of Dental Medicine, University of Porto, Rua Dr. Manuel Pereira da Silva, 4200-393 Porto, Portugal; 3 Departamento de Ciências da Vida, Centre for Functional Ecology, University of Coimbra (CFE-UC), Calçada Martim de Freitas, 3000-456 Coimbra, Portugal

**Keywords:** Forensic anthropology, Dental anomalies, Fluctuant asymmetry, Environment, Agenesis

## Abstract

Traditionally, dental identification techniques are used to establish identity or assist in reconstructing an individual's biological profile. However, other aspects of identity, namely socioeconomic status (SES), can be estimated through teeth. This work aims to evaluate the influence of SES on third molar agenesis in a Portuguese population. X-rays from 448 subjects (223 belonging to a high and 225 to a low socioeconomic status) were assessed and demographic data (age, sex) and dental history were registered. Frequencies and associations between the variables were analyzed using the chi-square test. For each group, differences between third molar agenesis were studied using the Wilcoxon test. The significance level was 5%. X-rays displaying at least one agenesis were more common in females (in both SES groups). Differences between socioeconomic status were found in female subjects' upper right and lower left third molars, with a higher frequency of agenesis in the lower SES group. Agenesis of lower third molars displayed fluctuant asymmetry in both groups, whereas agenesis of upper third molars was also present in the lower SES group. These results suggest that socioeconomic status can affect third molar agenesis prevalence, and fluctuant asymmetry seems more prevalent in the lower SES, as it affects all third molars.

## Introduction

In forensic human identification, we often need to reconstruct a person’s biological profile by estimating a) population affinity, b) sex, c) age, and d) height. For many of these techniques, reference values have been developed to guide the interpretation of each aspect of a person’s biological profile [1–3]. These reference values have been set using mainly samples from a low socioeconomic population and are used regularly, despite the individual socioeconomic group. Yet, this may be problematic since it is known that different socioeconomic groups may display important biological differences [2], namely in skeletal age [4]. For example, Carneiro et al. [5] studied a large sample of Portuguese children from different socioeconomic groups and found that patients from a higher socioeconomic status (SES) showed a consistent advancement in the maturation of the third molar. However, SES differences diminish and eventually disappear in the last stages of root maturation. In a similar sample, studying the mandibular first seven left teeth, Caldas and Cardoso [6] verified that low SES girls had advanced dental maturation. Low SES boys, however, showed more often a delayed maturation relative to their high SES counterparts. Still, this pattern was inconsistent, and a clear socioeconomic difference seems absent in boys.

Hence, it would be helpful to develop methodologies that would allow us to estimate a person’s socioeconomic status (SES) in human forensic identification before performing data interpretation and choosing proper reference values.

Dental tissues are known to have extraordinary resistance [7]. Thus, techniques based on dental tissues may be of the utmost importance in human forensic identification, where other tissues may present severe decay and be, therefore, unsuitable for providing useful information.

SES can be related to oral health and dental treatments, as people from favored socioeconomic status usually display more expensive dental care treatments and have better oral condition [8–12]. However, these features can be misleading, as they only reflect environmental factors. Additionally, in some countries, dental care is available through public services, whereas in other places, the only option for dental treatment is private practice. Therefore, additional markers for determining socioeconomic status are needed.

The congenital absence of teeth is called agenesis. Agenesis is one of the most common dental abnormalities in humans and can occur as a single event or as a manifestation of a congenital syndrome [13]. Hypodontia and anodontia can describe congenital agenesis in cases of the absence of up to six teeth (excluding third molars) and for the absence of all teeth in both dentitions, respectively [14]. Third molar (3 M) agenesis is the most common dental number anomaly, and there is vast and highly contradictory literature on factors relating to its occurrence [15]. The contributions of environment and genetics to 3 M agenesis still need to be better understood. While some mutations have been shown to cause agenesis [16], environmental disturbances occurring before the initiation of 3 M mineralization have been shown to produce this anomaly as well [17].

Differences found between measurements of the left minus right side in bilateral features, such as the number of teeth, are called fluctuating asymmetry (FA) [18]. Some authors suggest that development instability, reflected by FA, is far more related to environmental stress than to changes in the mechanism underlying developmental homeostasis [18]. Conversely, it has been suggested that the expression of FA may be character-specific such that characters do not react similarly to genetic or environmental changes during development camp [19]. Furthermore, other authors suggest that FA among different characters has been detected primarily in the population rather than at an individual level [20].

This work aims to evaluate if FA in third molar agenesis is more prevalent in a sample of individuals from a low SES group than in a sample of a higher SES group.

## Materials and methods

X-rays from 448 subjects (223 high SES, 225 low SES) were evaluated for 3 M agenesis by a single experienced observer (IMC). Age ranged between 9 and 30 years (mean age = 16.73 years, standard deviation (SD) = 5.462). In the high SES, the mean age was 16.91 years (SD = 5.361), whereas, in the low, it was 16.56 years (SD = 5.566). The age distribution of the two subsamples according to SES and the entire sample is provided in Table 1.

**Table 1:** Age distribution according to sex and SES

	High SES			Low SES		
Age (years)	Female	Male	Total	Female	Male	Total
9	7	14	21	10	14	24
10	15	11	26	18	17	35
11	14	11	25	8	9	17
12	8	8	16	8	4	12
13	10	5	15	5	10	15
14	5	9	14	6	7	13
15	4	3	7	7	7	14
16	6	5	11	4	7	12
17	5	5	10	8	3	11
18	4	5	9	6	3	9
19	3	5	8	1	4	5
20	7	7	14	8	2	10
21	6	6	12	2	10	12
22	8	1	9	4	3	7
23	8	4	12	4	4	8
24	8	2	10	5	5	10
25	6	2	8	6	6	12
26	14	2	16	5	4	9
27	1	0	1	4	2	6
30	0	0	0	0	1	1

X-rays belonging to individuals assigned to the low SES group were provided by the Faculty of Dental Medicine, University of Porto (FMDUP) clinical services. In contrast, those individuals assigned to the high SES were provided by a private clinic also from Porto, Portugal. These X-rays were made for diagnostic purposes, and all participants signed an informed consent beforehand. Furthermore, this study was authorized by the Ethics Committee of the Faculty of Dental Medicine of the University of Porto (FMDUP Ethics Committee) as indicated in the approval statement number 8_1499.06.12.2012. Both sets of X-rays were part of an utterly anonymized database in which demographic data and medical and dental history were also recorded. Exclusion criteria were: age younger than 9 or older than 30, nationality other than Portuguese, any treatment performed to any 3 M, and presence of any condition disturbing normal tooth development. As for these conditions, we’ve considered endocrine disorders (such as thyroid or parathyroid disorders), cerebral palsy, hematologic disorders, celiac disease, genetic diseases/disorders, and metabolic syndromes, as they all seem to affect dental mineralization [21–34].

Individual assignations to SES groups were based on the individual’s wealth according to their income (indirectly) and the level of education (directly). Income was evaluated considering their access to dental health care. Note that, in Portugal, the National Health Service (NHS) is almost free of charge and available to everyone. However, oral health care is virtually non-existent in the NHS, and most dental treatment options exist only in private clinics (with or without health insurance plans associated) and university dental clinics. The latter offers all types of dental treatment, often carried out by supervised students, with a much lower budget; however, university dental clinics are often fully booked and accessible to only a few. We’ve assumed that people who attended a private clinic are more favored economically than those who attend the services of the FMDUP clinic. In addition, the sample from the private clinic included only individuals without health insurance (and therefore able -themselves or through their parents—to bear the total cost of dental treatment).

The level of education was evaluated (considering university education as higher education level). Thus, the individuals assigned to the high SES group included those with a higher education or, if a minor, with at least one parent with a higher education, attending a private practice, and not being holders of health insurance. Conversely, participants assigned to the low SES consisted of those without higher education or, if minors, those with parents lacked higher education, utilizing the clinical services of FMDUP.

All participants in this study were evaluated for 3 M agenesis and had their demographic data (age, sex, and SES) recorded. 3 M agenesis was evaluated confronting the patient’s dental history and the visualization of 3Ms in the orthopantomogram. If a 3 M was not referred to as extracted in the patient’s records and was not visible in the x-ray, it was considered an agenesis. All 3 M were evaluated to evaluate agenesis symmetry.

Data were analyzed using SPSS (Statistical Package for Social Sciences), version 27.0. Frequencies and associations between variables were assessed with chi-square tests. Differences between 3 M agenesis by side were studied for each group using the Wilcoxon test. The significance level established was 5%.

## Results

Almost half of the x-rays revealed agenesis of at least one 3 M (n = 227, 47.8%). In males, 102 (46.4%) had at least one agenesis, whereas, in females, agenesis was present in almost 50% of the cases (n = 125, 49%). X-rays displaying at least one agenesis were more common in the high SES group, both in females (n = 69, 55.2%) and males (n = 53, 52.0%), with no significant statistical differences between SES groups (p = 0.900 and p = 0.429).

Considering differences between sexes, x-rays from females displaying at least one congenitally absent 3 M were more common, regardless of their SES (high SES group: n = 69, 56.6%; low SES group: n = 56, 53.3%), with no significant statistical differences between sexes (p = 0.897 and p = 0.224).

When considering 3 M isolated, upper right 3 M (UR3M) agenesis was present in 135 individuals (30.13%), upper left third molar (UL3M) agenesis in 120 (26.79%), lower left third molar (LL3M) agenesis in 101 (22.54%), and (LR3M) agenesis in 29 (6.47%). Significant differences by SES groups were only found among females for the upper right (p = 0.008) and lower left 3 M (p = 0.044), with a higher frequency among females of the lower SES group (56.72%) relative to their high SES counterparts (35.51%). 3 M agenesis prevalence by SES and is presented in Table 2.

**Table 2:** 3 M agenesis according with SES and sex, n(%)

Teeth	Females			Males		
	High SES	Low SES	p value	High SES	Low SES	p value
UR3M	38 (35.51)	38 (56.72)	0.008	34 (38.20)	25 (37.88)	1
UL3M	40 (37.38)	28 (41.79)	0.633	30 (33.71)	22 (33.33)	1
LL3M	27 (25.23)	27 (40.30)	0.044	26 (29.21)	21 (31.82)	0.728
LR3M	9 (8.41)	4 (5.97)	0.768	11 (12.36)	5 (7.58)	0.428

Agenesis of lower 3 M displayed FA in both groups, despite the individual sex (p = 0.025 and p = 0.021, for the high and low SES groups, respectively). Agenesis of the upper 3 M displayed FA only in the lower SES group (p < 0.001) (Table 3).

**Table 3:** Differences in 3 M agenesis according to side, considering SES and sex – wilcoxon test

Teeth	Females		Males	
	High SES	Low SES	High SES	Low SES
UR3M –UL3M	-0.775 (0.439)	-2.309 (0.021)	-0.471 (0.637)	-3.207 (0.001)
LL3M-LR3M	-2.236 (0.025)	-4.041 (< 0.001)	-4.264 (< 0.001)	-5.516 (< 0.001)

## Discussion

SES can be defined as the position or relative order that an individual occupies in the hierarchy based on social and economic attributes, which can be reflected by access to differential resources and assets valued by society, such as nutrition, health care, or academic training camps [8]. Usually, SES is evaluated according to three main factors: level of education, income, and occupation. Level of education can be conceptualized as the age at which the studies ended, the number of years engaged in studies, the degree attained, or simply as a dichotomy between literacy and illiteracy. Income level limits direct access to the fulfilment of basic needs and may be related to the possibility of enjoying adequate housing, nutrition, or health care and, therefore, crucial for developing healthy lifestyle habits. As such, it is understood that access to healthcare differences may reflect SES [8]. It is further known that access to better nutrition and access to health care are among the most important changes in the environment experienced by members of different SES groups [35]. Thus, SES can mirror access to better nutrition and health.

Regarding occupation, the knowledge an individual possesses is, perhaps, more important, as this can affect behavior and change their lifestyle. This assessment is often preferred to the description of professional activity since this does not take into account individuals who are not part of the work society and is strongly linked with age, meaning that this factor may not be as important as the level of education [35]. Therefore, we decided to split our sample into different SES based on the level of education and evaluation of access to health care. We recognize that the system of assignment by SES status may have flaws. Still, it also provides a fair reflection of the differences in access to health care and the quality of life experienced by the participants of this study. Furthermore, this system of SES assignment has been used previously [5, 6, 8].

In our sample, we have chosen individuals between 9 and 30 years of age. These thresholds were defined based on the age which third molar development is expected to begin [15]; additionally, we have chosen individuals with a known dental, medical history, to be sure of the third molar absence nature (extraction vs. agenesis). We have found it very hard to control this in individuals over age 30, so we’ve defined the upper threshold for sample inclusion at this age.

Agenesis has been shown to have a genetic component [13, 36]. Still, it has been stated it can also develop as the result of developmental pathology [37], delayed growth [38], and the amount of space available in the jaw [39]. Additionally, being the last tooth to develop and erupt, M3 may be more sensitive to environmental perturbation [37], thus, potentially it can reflect early life history, and hence, be linked with the individual’s SES.

Theoretically, teeth number and morphology should be a symmetrical mirror image between antimeric teeth. Despite this, perturbations during development, such as environmental factors, may lead to deviations from symmetry, and therefore people more exposed to environmental hazards should display FA more frequently. Theoretically, people from a low SES are more prone to be exposed to environmental hazards, and if FA reflects, at least partially, developmental instability caused by environmental factors, FA present in 3 M agenesis may present itself as more prevalent in low SES groups.

Regarding our results, our sample displayed a very high frequency of 3 M agenesis (47.8%). A recent study conducted in two Mediterranean populations, from Italy and Lebanon, showed that 23.4% of the studied sample had at least one-third molar agenesis [40]. According to Carter and Worthington [15], the worldwide rate of 3 M agenesis was found to be 22.63%, with a 95% confidence interval between 20.64% to 24.76%, although the estimates ranged from 5.32% to 56.0%. Even if our numbers are inside the described worldwide range, our data points to a high prevalence of agenesis, and one possible explanation for our results may be linked to population differences. Rozkovcová and co-workers [41] reported large diversities regarding the frequency of agenesis of third molars in different populations from practically zero (Tasmania) to nearly 100% (Mexican Indians). Similarly, Carter and Worthington [15] also referred that 3 M agenesis develops independently in different populations. Interestingly, population differences in 3 M agenesis prevalence have been previously established, with the prevalence being highest among Sinodonts (Northeast Asians and Native Americans; 44%), followed by Southeast Asians (20.9%), and Europeans (14.5%), with agenesis being least common in Sub-Saharan Africans (0.5%) and Australian aboriginals (1.8%) [42, 43]. However, there are conflicting reports on this subject. For instance, Mani et al. [44] reported a rate of 3 M agenesis among 25.7% of children, almost half the value presented in other studies. Alam and co-workers [45] reported values of 3 M agenesis of 30% and 33% among Malaysian Malay and Chinese, respectively. Similarly, Celikoglu and Kamak [46] reported a 22.7% prevalence of 3 M agenesis as Reda et al. [40] reported a value of 23.4% in European and Mediterranean populations, respectively. These values, like our present results, don’t agree with those referred to previously.

Regarding 3 M agenesis sex distribution, females had a higher prevalence of agenesis, although this difference is not statistically significant; this was also referred to by other authors [47]. Conversely, other studies reported significant differences in 3 M agenesis with females displaying a higher frequency [13, 42, 43, 45, 46, 48, 49], and other authors reported male 3 M agenesis rates as being higher than those of females [50]. Again, population differences may explain these results.

Thus, the individual’s population affinity should be considered when interpreting 3 M agenesis.

In what concerns differences between SES groups, we’ve found statistically significant differences in females in teeth UR3M and LL3M (p = 0.008 and p = 0.044, respectively), with a higher prevalence of agenesis in the lower SES group (35.46% vs. 29.24%). We could not compare our results since we’ve found no data in the literature regarding the influence of SES in dental agenesis. However, it is recognized that SES can affect tooth development. Cruvinel et al. [51] referred to low birth weight as a risk factor for hypoplasia, which can be related to SES. We recognize that enamel hypoplasia and agenesis are not manifestations of the same medical conditions; nevertheless, one can assert that unfavorable socioeconomic status may limit or even preclude the development of specific structures, namely teeth. Massoni and co-workers [52] reported that poor socioeconomic conditions might be associated with low birth weight or nutritional risk and the development of body structures, particularly in the teeth since they are particularly vulnerable to structural changes during formation. Our results indicate that these disadvantages may be more important in females.

Regarding FA, we found 3 M agenesis asymmetry in the lower SES group in both U3M and L3M, whereas in the upper SES group, asymmetry was only found in the L3M.

Theoretically, tooth number and morphology should represent a symmetrical mirror image between antimeric teeth. Nevertheless, perturbations during development, such as environmental factors, may lead to deviations from symmetry. These deviations are known as fluctuating asymmetry. So, considering different SES groups, one can expect FA to be more common among individuals of low SES since, in theory, the members of this group are at a greater liability of being exposed to environmental hazards. This agrees with our findings that did, in fact, point to asymmetry in agenesis of U3M and L3M in the lower SES group, whereas in the upper SES group, asymmetry only happened in the L3M. In this manner, one can state that fluctuating asymmetry in U3M agenesis may point to a lower SES. This may also mean that the ontogeny process of U3M is more robust since fluctuating asymmetry of L3M is expected, regardless of the SES group.

## Conclusion

Our sample displayed a very high frequency of 3 M agenesis, suggesting the need to consider population data when assessing 3 M agenesis. The UR3M was the dental element most often congenitally absent. In females, UR3M and LL3M may point to a disadvantaged socioeconomic status. Fluctuating asymmetry in U3M is yet another factor that may help determine SES, pointing out an unfavorable SES. Conversely, fluctuating asymmetry of L3M is present in members of both SES.

## Key points

1. Socioeconomic status (SES) may impact skeletal and dental reference values.
2. Methodologies to estimate socioeconomic status (SES) would be useful in forensic human identification.
3. This work evaluated the influence of SES on third molar agenesis in a Portuguese population.
4. UR3M and LL3M agenesis in females and fluctuating asymmetry in U3M can be associated with low SES.
5. Third molar fluctuating asymmetry and agenesis may be valuable parameters in SES estimation.

## Data Availability

Data is available upon request.

## References

[1] Austin D, King RE. The biological profile of unidentified human remains in a forensic context. Acad Forensic Pathol. 2016;6(3):370–90. 31239913 10.23907/2016.039 PMC6474556

[2] Carneiro JL, et al. Is Demirjian’s original method really useful for age estimation in a forensic context? Forensic Sci Med Pathol. 2015;11(2):216–21. 25700829 10.1007/s12024-015-9656-x

[3] Perez-Mongiovi D, Teixeira A, Caldas IM. The radiographic visibility of the root pulp of the third lower molar as an age marker. Forensic Sci Med Pathol. 2015;11(3):339–44. 26105787 10.1007/s12024-015-9688-2

[4] Freitas D, et al. Skeletal maturity and socio-economic status in Portuguese children and youths: The Madeira growth study. Ann Hum Biol. 2004;31(4):408–20. 15513692 10.1080/03014460410001713050

[5] Carneiro JL, et al. Examining the socioeconomic effects on third molar maturation in a Portuguese sample of children, adolescents and young adults. Int J Legal Med. 2017;131(1):235–42. 27761650 10.1007/s00414-016-1476-3

[6] Caldas IM, Cardoso HFV. Socioeconomic differences in permanent teeth mineralization of Portuguese girls and boys from Porto. Portugal Anthropol Anz. 2021;78(4):267–77. 33595590 10.1127/anthranz/2021/1313

[7] Lacruz RS, et al. Dental enamel formation and implications for oral health and disease. Physiol Rev. 2017;97(3):939–93. 28468833 10.1152/physrev.00030.2016 PMC6151498

[8] Cardoso HF. A quantificação do estatuto socioeconómico em populações contemporâneas e históricas: dificuldades, algumas orientações e importância na investigação orientada para a saúde. Antropol Port. 2005;22:247–72.

[9] Guan Y, et al. Socioeconomic inequalities in dental caries among 5-year-olds in four Chinese provinces. Community Dent Health. 2015;32(3):185–9. 26513856

[10] Mathur MR, et al. Socioeconomic inequalities in dental caries and their determinants in adolescents in New Delhi, India. BMJ Open. 2014;4(12):e006391. 25500618 10.1136/bmjopen-2014-006391 PMC4267077

[11] Paula JS, Ambrosano GM, Mialhe FL. The impact of social determinants on schoolchildren’s oral health in Brazil. Braz Oral Res. 2015;29:1–9. 26313351 10.1590/1807-3107BOR-2015.vol29.0098

[12] Paula JS, et al. The influence of oral health conditions, socioeconomic status and home environment factors on schoolchildren’s self-perception of quality of life. Health Qual Life Outcomes. 2012;10:6. 22244092 10.1186/1477-7525-10-6 PMC3285522

[13] Vastardis H. The genetics of human tooth agenesis: new discoveries for understanding dental anomalies. Am J Orthod Dentofacial Orthop. 2000;117(6):650–6. 10842107

[14] Rathi NV, et al. Dental management of Rothmund-Thomson syndrome with partial anodontia. BMJ Case Rep. 2015. 10.1136/bcr-2015-209994 PMC4460316 26032705

[15] Carter K, Worthington S. Morphologic and demographic predictors of third molar agenesis: A systematic review and meta-analysis. J Dent Res. 2015;94(7):886–94. 25883107 10.1177/0022034515581644

[16] Shimizu T, Morita W, Maeda T. Genetic mapping of agenesis of the third molars in mice. Biochem Genet. 2013;51(9–10):728–36. 23736965 10.1007/s10528-013-9602-0

[17] Swee J, et al. Inferior alveolar nerve block and third-molar agenesis: a retrospective clinical study. J Am Dent Assoc. 2013;144(4):389–95. 23543693 10.14219/jada.archive.2013.0132

[18] GM C. Developmental Instability: Its Origins and Evolutionary Implications. 1 ed. Contemporary Issues in Genetics and Evolution. Temple, Arizona: Springer Dordrecht. 1994;441.

[19] Leary RF, Knudsen KL. Genetic, environmental, and developmental causes of meristic variation in rainbow trout. Acta Zoologica Fennica. 1992;191:79–95.

[20] Soule M, Baker B. Phenetics of natural populations IV. The population asymmetry parameter in the butterfly coenonympha tullia. Heredity. 1968;23(4):611–4. 5253588 10.1038/hdy.1968.78

[21] Staufer K, Hamadeh S, Gesch D. Failure of tooth eruption in two patients with cerebral palsy and bruxism-a 10-year follow-up: a case report. Spec Care Dentist. 2009;29(4):169–74. 19573044 10.1111/j.1754-4505.2009.00083.x

[22] Oliveira CA, et al. Bruxism control in a child with cerebral palsy. ISRN Dent. 2011;2011:146915. 21991456 10.5402/2011/146915 PMC3170074

[23] Hazza’a AM, Al-Jamal G. Dental development in subjects with thalassemia major. J Contemp Dent Pract. 2006;7(4):63–70. 16957792

[24] Pithon MM, Ruellas CV, Ruellas AC. Orthodontic treatment of a patient with Type 1 diabetes mellitus. J Clin Orthod. 2005;39(7):435–9. 16100417

[25] Mendes PH, et al. Orofacial manifestations in patients with sickle cell anemia. Quintessence Int. 2011;42(8):701–9. 21842010

[26] Condo R, et al. The dental age in the child with coeliac disease. Eur J Paediatr Dent. 2011;12(3):184–8. 22077688

[27] Lee W, O’Donnell D. Severe gingival hyperplasia in a child with I-cell disease. Int J Paediatr Dent. 2003;13(1):41–5. 12542623 10.1046/j.1365-263x.2003.00419.x

[28] Wendling D, et al. Adult hypophosphatasia. Current aspects Joint Bone Spine. 2001;68(2):120–4. 11324927 10.1016/s1297-319x(00)00238-4

[29] Mornet E, Nunes ME. Hypophosphatasia, in GeneReviews, R.A. Pagon, et al., Editors. 1993: Seattle (WA).

[30] Antunes LA, et al. Dental findings and oral health status in patients with mucopolysaccharidosis: a case series. Acta Odontol Scand. 2012. 10.3109/00016357.2011.654255 22376155

[31] McGovern E, et al. Oral features and dental health in Hurler Syndrome following hematopoietic stem cell transplantation. Int J Paediatr Dent. 2010;20(5):322–9. 20545789 10.1111/j.1365-263X.2010.01055.x

[32] Collins MA, et al. Dental anomalies associated with amelogenesis imperfecta: a radiographic assessment. Oral Surg Oral Med Oral Pathol Oral Radiol Endod. 1999;88(3):358–64. 10503869 10.1016/s1079-2104(99)70043-0

[33] Martelli-Junior H, et al. Amelogenesis imperfecta and nephrocalcinosis syndrome: a case report and review of the literature. Nephron Physiol. 2011;118(3):62–5. 10.1159/000322828 21212699

[34] Suri L, Gagari E, Vastardis H. Delayed tooth eruption: pathogenesis, diagnosis, and treatment. A literature review. Am J Orthod Dentofacial Orthop. 2004;126(4):432–45. 15470346 10.1016/j.ajodo.2003.10.031

[35] Cardoso HF, Heuze Y, Julio P. Secular change in the timing of dental root maturation in Portuguese boys and girls. Am J Hum Biol. 2010;22(6):791–800. 20721978 10.1002/ajhb.21084

[36] Garn SM, Lewis AB. The relationship between third molar agenesis and reduction in tooth number. Angle Orthod. 1962;32(1):14–8.

[37] Shapira J, Chaushu S, Becker A. Prevalence of tooth transposition, third molar agenesis, and maxillary canine impaction in individuals with down syndrome. Angle Orthod. 2000;70(4):290–6. 10961778 10.1043/0003-3219(2000)070<0290:POTTTM>2.0.CO;2

[38] de Castro JMB. Third molar agenesis in human prehistoric populations of the Canary Islands. Am J Phys Anthropol. 1989;79(2):207–15. 2662782 10.1002/ajpa.1330790208

[39] Kajii TS, et al. Agenesis of third molar germs depends on sagittal maxillary jaw dimensions in orthodontic patients in Japan. Angle Orthod. 2004;74(3):337–42. 15264644 10.1043/0003-3219(2004)074<0337:AOTMGD>2.0.CO;2

[40] Reda B, et al. Radiographic evaluation of non-syndromic third molar agenesis in two Mediterranean populations. Med Pharm Rep. 2021;94(3):353–7. 34430858 10.15386/mpr-1914 PMC8357356

[41] Rozkovcová E, Marková M, Dolejsí J. Studies on agenesis of third molars amongst populations of different origin. Sb Lek. 1999;100(2):71–84. 11220165

[42] Irish JD. Characteristic high- and low-frequency dental traits in sub-Saharan African populations. Am J Phys Anthropol. 1997;102(4):455–67. 9140538 10.1002/(SICI)1096-8644(199704)102:4<455::AID-AJPA3>3.0.CO;2-R

[43] Turner CG 2nd. Late Pleistocene and Holocene population history of East Asia based on dental variation. Am J Phys Anthropol. 1987;73(3):305–21. 3303958 10.1002/ajpa.1330730304

[44] Mani SA, Mohsin WS, John J. Prevalence and patterns of tooth agenesis among Malay children. Southeast Asian J Trop Med Public Health. 2014;45(2):490–8. 24968691

[45] Alam MK, et al. Multivariate analysis of factors affecting presence and/or agenesis of third molar tooth. PLoS ONE. 2014;9(6): e101157. 24967595 10.1371/journal.pone.0101157 PMC4072760

[46] Celikoglu M, Kamak H. Patterns of third-molar agenesis in an orthodontic patient population with different skeletal malocclusions. Angle Orthod. 2012;82(1):165–9. 21815812 10.2319/041911-274.1 PMC8881022

[47] Celikoglu M, Miloglu O, Kazanci F. Frequency of agenesis, impaction, angulation, and related pathologic changes of third molar teeth in orthodontic patients. J Oral Maxillofac Surg. 2010;68(5):990–5. 20096980 10.1016/j.joms.2009.07.063

[48] Bansal S, et al. Frequency of impacted and missing third molars among orthodontic patients in the population of Punjab, Indian. J Oral Sci. 2012;3:24.

[49] Carvalho S, Mesquita P, Afonso A. Prevalência das anomalias de número numa população portuguesa. Estudo radiográfico. Revista Portuguesa de Estomatología, Medicina Dentária e Cirugia Maxilofacial. 2011;52(1):7–12.

[50] Upadhyaya C, et al. Agenesis of third molars in orthodontic patients attending dhulikhel hospital. Orthod J Nepal 2013;2.

[51] Cruvinel VR, et al. Prevalence of enamel defects and associated risk factors in both dentitions in preterm and full term born children. J Appl Oral Sci. 2012;20(3):310–7. 22858696 10.1590/S1678-77572012000300003 PMC3881774

[52] Massoni AC, et al. Socioeconomic factors, nutritional risk, and enamel defects in children from João Pessoa, Paraíba State, Brazil. Cad Saude Publica. 2007;23(12):2928–37. 18157335 10.1590/s0102-311x2007001200014

